# Caging and Uncaging Genetics

**DOI:** 10.1371/journal.pbio.1002525

**Published:** 2016-07-26

**Authors:** Tom J. Little, Nick Colegrave

**Affiliations:** University of Edinburgh, Institute of Evolutionary Biology, School of Biological Sciences, Edinburgh, United Kingdom

## Abstract

It is important for biology to understand if observations made in highly reductionist laboratory settings generalise to harsh and noisy natural environments in which genetic variation is sorted to produce adaptation. But what do we learn by studying, in the laboratory, a genetically diverse population that mirrors the wild? What is the best design for studying genetic variation? When should we consider it at all? The right experimental approach depends on what you want to know.

## Abstract 2

Experiments on a single genotype are powerful and appropriate. Experiments on multiple genotypes are powerful and appropriate. The right experimental design depends on the question being asked.

## Introduction

Biologists of all kinds have the option of using defined genetic lines for experiments, for example, inbred lines of mice and rats, nematodes, fruit flies, or *Arabidopsis* stocks. An alternative is the use of outbred stocks, and, indeed, subject areas with especially strong traditions in laboratory experimentation have been encouraged to pay greater attention to the study of wild populations [1–3]. Alongside this, we frequently hear calls for the expansion of experimental designs to include more genotypes [4] or to place these genotypes in a wider range of environments [5]. One advocated goal in all of this is to add an extra dose of realism to our science to better understand how our treatments, or particular biological phenomena, might manifest in a more natural context. There is potential to misinterpret this point. Although calls for realism are typically heard within a particular context, the range of contexts spans much of biology, for example, immunology [6], toxicology [7,8], host–parasite interactions [9–11], or coevolution and the selective maintenance of breeding systems [12]. Thus, at stake are very general issues regarding why biologists choose a particular experimental design, and the question becomes: What is gained and lost in a laboratory study of, at one extreme, an outbred population compared to, at the other extreme, the study of a single genotype? Here, we synthesize these ideas, highlighting the pros and cons of the various ways experiments can incorporate genetic variation.

## In Defence of One

The essence of an experiment is reduction in the number of explanatory variables. Experimental control of environments leads to less variation within treatments, improving our chances of finding differences between treatments. As a simple example, we would not want to study the effect of a treatment on plants held in a faulty incubator whose lights flickered on and off randomly throughout the day and whose temperature varied spatially inside. A properly functioning incubator would eliminate photoperiod as a source of variation and limit environmental heterogeneity amongst our experimental plants, improving the power of our experiment.

**Fig 1:**
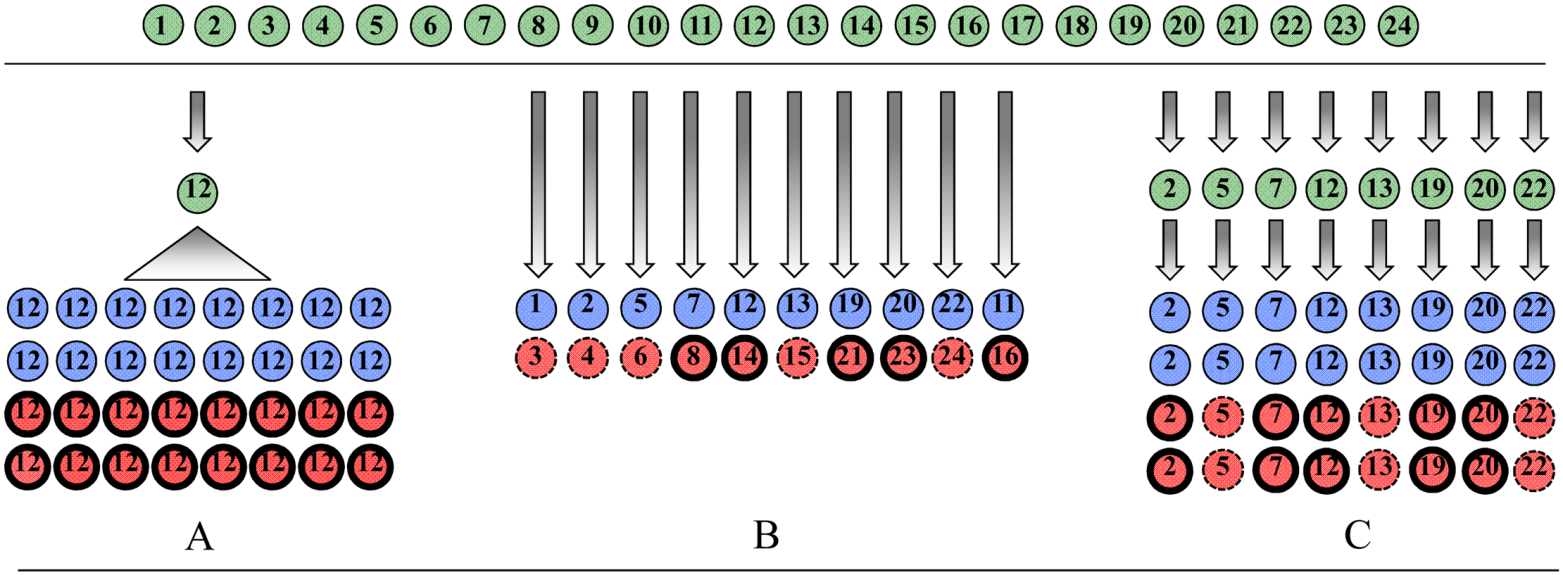
A hypothetical outbred population of 24 genotypes to be sampled and studied in the laboratory to determine the effect of a particular treatment: for example, exposure to a heat shock. Red represents the heat-shock treatment and blue is the control. Half of the genotypes can be expected to respond positively to the treatment by growing a thicker carapace (thick lines), while the other half are expected to respond by growing a thinner carapace (dashes). Here are three possible study designs: **A)** Replicated single genotype studies have the most power to detect a treatment effect, albeit only in one direction. If, for example, we are primarily interested in the biological consequences of a thicker carapace, then (A) is the optimal study design. **B)** A random sample of the outbred population cannot detect genetic effects and may not even detect a treatment effect if the negative trait values are similar in magnitude to the positive ones. Increasing the number of genotypes will not help. The experiment represented by (B) does a good job of representing the population average effect, though this may be of little value if the research is interested in carapace thickening in response to heat shock. **C)** A set of eight replicated inbred lines can reveal genetic variation and detect a treatment effect by revealing treatment by genotype effects.

Thus, in many cases, if the aim of a study is to test the effect of a treatment, then the most effective approach is to make use of organisms that are as genetically uniform as is practicable (Fig 1A). A single inbred line, or an ameiotic clone, is ideal. The reasoning behind this is simple: in any experiment, we are trying to separate variation caused by our treatment from variation due to other sources. If we carry out a study on genetically diverse subjects, then genetic variation for the character of interest, just like a faulty incubator, can make it more difficult for us to detect the treatment effects in which we are interested. Working with a single genotype removes this source of variation, maximising our power to detect effects of treatments. Thus, the argument for carrying out experimental studies on a single genotype is simply a subset of the argument for using controlled laboratory conditions: we wish to reduce variation from sources in which we are not currently interested. We also often have the opportunity to choose genotypes. For example, if doing a behavioural experiment, one would not choose a strain for which the behaviour of interest had been bred out. Adding genotypes, some of which show a different expression of the trait, can reduce our capacity to study the trait of interest.

The success of this approach cannot be understated. The nematode *Caenorhabditis elegans* was initially isolated from a single source and, thus, is essentially a single genotype. The vast majority of laboratory *Escherichia coli* are descendants of one of two isolates. For decades, in the absence of noise-making genetic variation, laboratory models have contributed immensely to our understanding of developmental biology, signalling pathways, meiosis, ageing, adaptation, and phenotypic plasticity, among many other important biological phenomena. One reason this has been achieved, aside from the powerful features and malleability of these organisms, is that defined lines can be shared and studies replicated in different laboratories. There is no question genetically uniform model organisms have proven generalisable to other environments, other genotypes of the same species, and, indeed, across large phylogenetic distances.

## Genetic Variation and Outbred Populations

Model organisms have also contributed to our understanding of the effects of genotype on phenotype, particularly where we have crafted lines that differ at only a single locus. In these cases, we study genetic variation, but in the simplest possible format in the tradition of experimental biology. Expanding to more genetic variation might involve incorporating a set genotypes initially isolated, for example, from a wild population but then maintained as separate, often inbred and uniform, stocks in the lab. At the farthest end of the spectrum, we might even perform our experiment on an outbred population, which will contain much uncharacterised genetic diversity. The latter fosters understanding of how wild populations might respond to a treatment, yet it comes with important limitations. At the very least, if this genetic variation affects the character in which we are interested, then its presence in our study will add noise, making any treatment effects more difficult to detect. And, even if issues of reduced power can be minimised by increased sampling, if different genotypes respond to the experimental treatment in different ways (there is a genotype by treatment interaction), the average effect of a treatment on a diverse uncharacterized population may lead to misleading conclusions. Consider, in the extreme, a particular treatment that has an effect on the character in which you are interested, but in half of the genotypes in your population the effect is positive, whilst in the other half the effect is negative by the same amount (Fig 1B). An experiment on a diverse population will see no overall effect of the treatment. In contrast, if we carry out the experiment using a replicated set of genetically uniform lines, strains, or clones, we will discover strong effects that vary amongst genotypes (Fig 1C). By essentially repeating our experiment on this set of genetically uniform lines (Fig 1C), not only do we benefit from the increased statistical power that comes with reduced variation (compared to an outbred population), a comparison of treatment effects among lines gives us a measure of how variable any treatment effects will be in a genetically diverse population. When experimenting on an outbred population, we can say nothing about how treatment effects vary with genotype (genotype–environment interaction [GxE]) unless we have pedigree information.

GxE interactions are common, and they can be viewed as an argument against the use of single genotypes in experimental work [4,13]. The conclusions of single genotype studies are limited to that specific genotype, so the argument goes, and it is only by experimenting on genetically diverse material that we can generalise to the diverse populations that we care about. However, such an argument confuses the population to which the statistical conclusions can be formally applied (which is indeed the genotype) with the population to which the biological conclusions might be applied, which might be the genotype or the population it came from, or the species and beyond depending on the biological trait with which we are concerned. And, of course, concerns about the range of conditions over which our conclusions might apply are not restricted to genetic effects. The experiment might also not generalise to temperatures other than those studied, to different food types, another lab, or to a different country or planet. The statistical conclusions of single environment studies are limited to that specific environment or the specific range of treatments studied. For example, an experiment that compared phenotypes at 10°C and 20°C can, technically, only draw conclusions about these specific temperatures, yet we are often comfortable generalising such results to draw conclusions about the consequences of living at lower versus higher temperatures [14].

Thus, we need to carefully consider what we are trying to understand with an experiment and what we might be trying to generalise to when we add genotypes to our experiment. By adding many genotypes to our experiment, in addition to the obvious consequence that we can evaluate genetic variation (if we replicate genotypes), we also may gain a better understanding of the average response of a population. Understanding population average responses may be the goal of some ecologists, epidemiologists, or evolutionary biologists. For example, we might have an interest in the keystone species of an ecosystem from which we harvest, or in particular pathogens we want to control. In many other cases, however, we are not interested in our study species—our study species is a model for a trait of interest. If the extra genotypes merely get us closer to understanding our study species, we are not substantially closer to generalising beyond our species because variation between species is often much larger than variation within species. It is intriguing that many scientists are prepared to accept mice as a model for human immunity (i.e., to generalise across species, from mice to humans, in research of immense health and welfare importance), yet would quibble about where studies on one genotype might not generalise to other genotypes of the same species. Imagine that work on the *C*. *elegans* N2 strain had been rejected by the scientific community for fear its patterns would be limited to that strain. Similarly, Lenski’s long-term study of bacterial evolution [15] began with a single bacterial cell—an elegant design choice from which we gladly draw conclusions we hope will apply to other bacteria and far beyond.

## Concluding Remarks

When creating laboratory lines, a few issues need to be considered. First, although the study of defined lines fosters comparisons of results from different labs and, thus, extensive repetition of experiments, the maintenance of the original “wild-type” in different laboratories can generate significant divergence between these “replicate” wild-types, as well as between laboratory strains and new field isolates. Second, the process of establishing lines in the laboratory often involves inbreeding, and inbred lines may suffer increased developmental instability, which is not a source of variation in which we are likely to be interested. Moreover, inbreeding generates homozygosity across much of the genome, which does not capture certain genetic effects that wild genotypes almost invariably experience. On the flip side, inbreeding reveals the phenotypic effect of recessive mutations, which has been a tremendously powerful tool in biology.

Establishing lines from wild stock may also involve selection. A salient example concerns the effect of caloric restriction on ageing. Caloric restriction has been shown to lead to longer life, but this observation appears to be more prevalent in long-term laboratory lines than in wild populations. It has been speculated that some model organisms have been selected for enhanced fecundity, which trades off with longevity, and caloric restriction has a much larger effect in this situation than in wild lines that have not been strongly selected for early reproduction [16]. Some laboratory animals have probably also been selected for depressed immune responsiveness, which reduces problems of autoimmunity in low infection laboratory environments [3], but compromises our ability to understand natural immune responses.

Thus, a question that arises in laboratory comparisons of how different genotypes respond to a treatment is: “Does a potentially unusual set of laboratory genotypes tell us more about the genetic variation basis of performance than an outbred population?” This issue is similar to the generalisation concern for single clone experiments. Laboratory populations inevitably misrepresent their wild friends to some degree (analogously, this issue burdens the establishment of stabilised cell lines). The worry that our laboratory organisms are a little odd must be balanced against the benefits of choosing the most powerful design for the question we have. We suggest that confounding variables—inbreeding depression or laboratory selection—are topics for valuable, but separate, downstream studies. This should be acceptable to us if we are prepared to use mice as a model for the human immune system or if we are encouraged by the flood of generalisable biological data that organisms such as *C*. *elegans* or *Drosophila* have provided.
